# Supplementary material for: Widespread temporal niche partitioning in an adaptive radiation of cichlid fishes
Source: Nat Ecol Evol. 2025 Aug 27;9(10):1938–50. doi: 10.1038/s41559-025-02819-z (PMC12507676; doi:10.1038/s41559-025-02819-z)
Supplement: Supplementary file 1 — Supplementary combined methods and results. [file 41559_2025_2819_MOESM1_ESM.pdf]

nature ecology & evolution

Supplementary information https://doi.org/10.1038/s41559-025-02819-z

Widespread temporal niche partitioning in an adaptive radiation of cichlid fishes

In the format provided by the authors and unedited

Supplementary Information for "Widespread temporal niche partitioning in an adaptive radiation of cichlid fishes"

Authors:
Annika L. A. Nichols[1+], Maxwell E. R. Shafer[1,2*+], Adrian Indermaur[3], Attila Rüegg[3], Rita Gonzalez-Dominguez[1], Milan Malinsky[3,4], Carolin Sommer-Trembo[3,5], Laura Fritschi[3], Amelia Mesich[2‡], Ayasha Abdalla-Wyse[2‡], Walter Salzburger[3], Alexander F. Schier[1]

Affiliations:
1 Biozentrum, University of Basel, Switzerland
2 Department of Cell and Systems Biology, University of Toronto, Canada (present address)
3 Zoological Institute, Department of Environmental Sciences, University of Basel, Switzerland
4 Department of Biology, Institute of Ecology and Evolution, University of Bern, Switzerland
5 Department of Paleontology, University of Zurich, Switzerland
*corresponding author
+ and ‡ equal-contribution

This PDF includes:

- **Combined Supplementary Methods/Results**
- **Supplementary References**

COMBINED SUPPLEMENTARY METHODS/RESULTS

The genetic signatures of activity patterns are complex and differ between clades

To investigate the genetic basis of temporal activity patterns and total rest, we followed an approach used in a recent study on the genetic basis of exploratory behaviour in Lake Tanganyika cichlids[1]. Briefly, we used a combination of a standard GWAS generalised linear model (GLM) and phylogenetic generalised least squares (pGLS) to account for phylogenetic relationships. This approach allows us to detect alleles associated with each trait across species that either predate the radiation and were differentially inherited in different lineages, or have been shared across species due to hybridization. We used cutoffs for genome-wide significance based on mutational simulations that should enrich for variants whose association with our behavioural traits is likely due to natural selection. Because of the relatively long history of the Tanganyikan cichlid radiation, and the deep ancestry of the species used in our study, recombination has broken down linkage between most variants in our dataset, and each single nucleotide polymorphism (SNP) is likely to provide an independent signature[1]. We focused on SNPs compared to insertions or deletions (INDELs) due to the potential ease in interpretation of SNPs, and the increased difficulty in accurately calling INDELs. We used 120 whole genome sequences from 60 species to look for association between ~39 million SNPs and (1) day-night preference (PC1 loadings), (2) preference for crepuscular activity (PC2 loadings), and (3) total amount of rest.

Selection and analysis of highly associated variants (HAVs)

We next wanted to identify SNPs whose allele frequencies were strongly or significantly associated with temporal activity preferences and total rest. Our study is potentially underpowered to identify significant associations between traits and allele frequencies using multiple-testing correction, due to the low number of species (60), and the high number of tested alleles (~40 million). However, a previous study has suggested that selecting the top fraction of SNPs that correspond to the lowest p-values in GLM and pGLS tests enriches for associations that are unlikely due to natural processes in the absence of selection[1]. Accordingly, we decided to focus on the top 99.99th percentile of SNPs with the lowest p-values for each association test (GLM and pGLS) and for each trait. This resulted in 766 unique SNPs that were highly associated with diurnal-nocturnal preference (PC1 loadings), 774 for crepuscular preference (PC2 loadings), and 752 for total rest. For each highly associated variant (HAV), we determined which allele (reference or alternative) was associated with nocturnal preferences (high PC1 loadings), crepuscular preference (high PC2 loadings), or total amounts of rest using the coefficients of the GLM models in R. These were then used for plotting allele frequencies, and to determine clusters of SNPs with distinct patterns across our species using hierarchical clustering and the function hclust in R.

The above approach allowed us to identify 766 highly associated variants (HAVs) for day-night preference, 774 HAVs for crepuscularity, and 752 HAVs for total rest that passed our genome wide significance threshold (**Supplemental Data 3**, **Fig. 3 and Extended Data Fig. 8**). HAVs were distributed evenly across the genome, and no overlap was observed between HAVs for different behaviours (**Extended Data Fig. 9**). Individual HAVs demonstrated clear association with our behavioural traits, including HAV NC_031975:1851366, which was found in species with strong nocturnal preference across tribes (e.g. *N. tretocephalus* [Neotre], *N. toae* [Neotoa], *O. boops* [Ophboo], *Xenotilapia spilopterus* [Xenspi], and *X. bathyphilus* [Xenbat]), but not in those with strong diurnal preferences (**Fig. 3a**).

To more systematically interrogate these patterns, we determined the directionality of the association between each allele and each behaviour, and clustered HAVs based on the frequency of whichever allele (reference or alternative) associated with positive PC1 loading scores (categories i-v, **Fig. 3b**), positive PC2 loading scores (**Extended Data Fig. 8a**), and high amounts of total rest (**Extended Data Fig. 8b**). For example, many nocturnal-associated alleles were mostly found within the Lamprologini (e.g. HAV NC_031971:2481009) (**Fig. 3a**), or mostly within the Ectodini (e.g. HAV NC_031982:23856121) (**Fig. 3a**). Additionally, there were alternative alleles which were present in the majority of species, and where the reference allele was associated with strong diurnality, including *N. buescheri*, which is one of the most strongly diurnal species in our study (e.g. HAV NC_031978:6846904, **Fig. 3a**). Similar trends were observed for HAVs associated with crepuscularity and total rest, with alleles shared across the radiation (category *iv*), alleles specific or private to certain tribes (e.g. Lamprologini or Ectodini) (categories *i*, *ii*, and *v*), as well as alleles whose absence was associated with the trait (**Extended Data Fig. 8**) (category *iii*, *v*). These results suggest that there is stratification of alleles, and that different alleles might underlie temporal activity patterns in different species or tribes. Together, these analyses suggest that the genetics of temporal activity patterns in cichlids are complex.

Gene ontological analysis of genes associated with HAVs

We next sought to identify genes that might be in proximity to the identified highly associated variants, and therefore may underlie temporal activity preferences or total rest. To annotate each SNP we used snpEff (version 5.2)[2] and a genome database built from the *O. niloticus* UMD NMBU genome assembly downloaded from the NCBI RefSeq database. Human orthologs for *O. niloticus* genes were identified using the NCBI datasets command line tools (https://github.com/ncbi/datasets), as well using Ensembl biomart (Ensembl release 111). To identify gene categories that were enriched we used the software SNP2GO, which annotates all SNPs with GO terms based on their proximity to genes, and therefore allows testing the enrichment for SNPs that affect particular GO terms. We used modified versions of the function snp2go, which allowed the testing of disease and tissue expression associated gene sets collected using the packages PhenoExam (version 0.1)[3] and RDAVIDWebService (version 1.28.0)[4] in R.

REFERENCES

1. Sommer-Trembo, C. *et al.* The genetics of niche-specific behavioral tendencies in an adaptive radiation of cichlid fishes. *Science* **384**, 470–475 (2024).
2. Cingolani, P. *et al.* A program for annotating and predicting the effects of single nucleotide polymorphisms, SnpEff: SNPs in the genome of Drosophila melanogaster strain w1118; iso-2; iso-3. *Fly (Austin)* **6**, 80–92 (2012).
3. Cisterna, A. *et al.* PhenoExam: gene set analyses through integration of different phenotype databases. *BMC Bioinformatics* **23**, 567 (2022).
4. Fresno, C. & Fernández, E. A. RDAVIDWebService: a versatile *R* interface to DAVID. *Bioinformatics* **29**, 2810–2811 (2013).
