# Supplementary material for: Widespread temporal niche partitioning in an adaptive radiation of cichlid fishes
Source: Nat Ecol Evol. 2025 Aug 27;9(10):1938–50. doi: 10.1038/s41559-025-02819-z (PMC12507676; doi:10.1038/s41559-025-02819-z)
Supplement: Supplementary file 2 — Reporting Summary [file 41559_2025_2819_MOESM2_ESM.pdf]

nature portfolio

Corresponding author(s): Maxwell E. R. Shafer

Last updated by author(s): Jun 18, 2025

Reporting Summary

Nature Portfolio wishes to improve the reproducibility of the work that we publish. This form provides structure for consistency and transparency in reporting. For further information on Nature Portfolio policies, see our Editorial Policies and the Editorial Policy Checklist.

Statistics

For all statistical analyses, confirm that the following items are present in the figure legend, table legend, main text, or Methods section.

n/a	Confirmed	
☐	☒	The exact sample size (*n*) for each experimental group/condition, given as a discrete number and unit of measurement
☐	☒	A statement on whether measurements were taken from distinct samples or whether the same sample was measured repeatedly
☐	☒	The statistical test(s) used AND whether they are one- or two-sided<br>*Only common tests should be described solely by name; describe more complex techniques in the Methods section.*
☐	☒	A description of all covariates tested
☐	☒	A description of any assumptions or corrections, such as tests of normality and adjustment for multiple comparisons
☐	☒	A full description of the statistical parameters including central tendency (e.g. means) or other basic estimates (e.g. regression coefficient) AND variation (e.g. standard deviation) or associated estimates of uncertainty (e.g. confidence intervals)
☐	☒	For null hypothesis testing, the test statistic (e.g. *F*, *t*, *r*) with confidence intervals, effect sizes, degrees of freedom and *P* value noted<br>*Give P values as exact values whenever suitable.*
☒	☐	For Bayesian analysis, information on the choice of priors and Markov chain Monte Carlo settings
☒	☐	For hierarchical and complex designs, identification of the appropriate level for tests and full reporting of outcomes
☐	☒	Estimates of effect sizes (e.g. Cohen's *d*, Pearson's *r*), indicating how they were calculated

Our web collection on statistics for biologists contains articles on many of the points above.

Software and code

Policy information about availability of computer code

Data collection	Scripts for recording and tracking were written in python and are available online (https://github.com/annnic/cichlid-tracking). All scripts for performing quality control and running analysis of behavioural activity, including generation of plots of cichlid weekly and daily speeds were written in python and are available online (https://github.com/annnic/cichlid-analysis).
Data analysis	Scripts for analysis of eco-morphological data, construction of phylogenetic plots, highly associated variant analysis, and gene ontology analysis were written in R and available online (https://github.com/maxshafer/cichlid_sleep_gwas). Scripts for running genome wide association analysis, including the GATK python, generation of genome masks, and variant identification and filtering were written in bash and available online (https://github.com/maxshafer/cichlid_sleep_gwas).

For manuscripts utilizing custom algorithms or software that are central to the research but not yet described in published literature, software must be made available to editors and reviewers. We strongly encourage code deposition in a community repository (e.g. GitHub). See the Nature Portfolio guidelines for submitting code & software for further information.

Data

Policy information about availability of data

All manuscripts must include a data availability statement. This statement should provide the following information, where applicable:

- Accession codes, unique identifiers, or web links for publicly available datasets
- A description of any restrictions on data availability
- For clinical datasets or third party data, please ensure that the statement adheres to our policy

The time-calibrated species tree, morphology and stable carbon (C) and nitrogen and (N) isotope signatures were taken from Ronco et al. 20215 (data available on Dryad: https://datadryad.org/stash/dataset/doi:10.5061/dryad.9w0vt4bbf). Raw data from cichlid activity tracking is available as a supplemental file associated with this submission. The unbinned behavioural tracks are available on Dryad (https://datadryad.org/dataset/doi:10.5061/dryad.j0zpc86sv). Results from GWAS studies are available as supplemental file associated with this submission.

Research involving human participants, their data, or biological material

Policy information about studies with human participants or human data. See also policy information about sex, gender (identity/presentation), and sexual orientation and race, ethnicity and racism.

Reporting on sex and gender	*Use the terms sex (biological attribute) and gender (shaped by social and cultural circumstances) carefully in order to avoid confusing both terms. Indicate if findings apply to only one sex or gender; describe whether sex and gender were considered in study design; whether sex and/or gender was determined based on self-reporting or assigned and methods used.<br>Provide in the source data disaggregated sex and gender data, where this information has been collected, and if consent has been obtained for sharing of individual-level data; provide overall numbers in this Reporting Summary. Please state if this information has not been collected.<br>Report sex- and gender-based analyses where performed, justify reasons for lack of sex- and gender-based analysis.*
Reporting on race, ethnicity, or other socially relevant groupings	*Please specify the socially constructed or socially relevant categorization variable(s) used in your manuscript and explain why they were used. Please note that such variables should not be used as proxies for other socially constructed/relevant variables (for example, race or ethnicity should not be used as a proxy for socioeconomic status).<br>Provide clear definitions of the relevant terms used, how they were provided (by the participants/respondents, the researchers, or third parties), and the method(s) used to classify people into the different categories (e.g. self-report, census or administrative data, social media data, etc.)<br>Please provide details about how you controlled for confounding variables in your analyses.*
Population characteristics	*Describe the covariate-relevant population characteristics of the human research participants (e.g. age, genotypic information, past and current diagnosis and treatment categories). If you filled out the behavioural & social sciences study design questions and have nothing to add here, write "See above."*
Recruitment	*Describe how participants were recruited. Outline any potential self-selection bias or other biases that may be present and how these are likely to impact results.*
Ethics oversight	*Identify the organization(s) that approved the study protocol.*

Note that full information on the approval of the study protocol must also be provided in the manuscript.

Field-specific reporting

Please select the one below that is the best fit for your research. If you are not sure, read the appropriate sections before making your selection.

☒ Life sciences ☐ Behavioural & social sciences ☐ Ecological, evolutionary & environmental sciences

For a reference copy of the document with all sections, see nature.com/documents/nr-reporting-summary-flat.pdf

Life sciences study design

All studies must disclose on these points even when the disclosure is negative.

Sample size	Up to 14 adult individuals per species were tracked (average 9 individuals, with a range from 2-14 see Supplementary Data 1). A number of n = 12 is sufficient to correctly infer a 50% difference in the means of two groups with a relatively high standard deviation (1 SD = 43% of the mean) (80% power, 5% type I error). We expected that many of the parameters we measured through video tracking will require such power, as often behavioural phenotypes are quite variable between individuals, or will have small differences in mean measurements. Therefore, we chose to phenotype up to 14 individuals per species, or the maximum number of individuals available to us through breeding or purchase.
Data exclusions	Tracking for animals which became sick, died or escaped were excluded.
Replication	Given the large scale design and nature of our study, we did not incorporate mechanisms to measure the reproducibility of our findings. In some case, and for certain species, we were able to replicate their activity pattern, and in all of those cases our results were reproducible (for example, when one species was tested over two separate weeks, these results could be compared).

Randomization	Randomization was not relevant for our study design
Blinding	Experimenters were not blinded during data collection, however, most data analysis was done in an automated manner (online tracking of fish behaviour), and therefore blinding was not necessary.

Reporting for specific materials, systems and methods

We require information from authors about some types of materials, experimental systems and methods used in many studies. Here, indicate whether each material, system or method listed is relevant to your study. If you are not sure if a list item applies to your research, read the appropriate section before selecting a response.

Materials & experimental systems

n/a	Involved in the study
☒	☐ Antibodies
☒	☐ Eukaryotic cell lines
☒	☐ Palaeontology and archaeology
☐	☒ Animals and other organisms
☒	☐ Clinical data
☒	☐ Dual use research of concern
☒	☐ Plants

Methods

n/a	Involved in the study
☒	☐ ChIP-seq
☒	☐ Flow cytometry
☒	☐ MRI-based neuroimaging

Animals and other research organisms

Policy information about studies involving animals; ARRIVE guidelines recommended for reporting animal research, and Sex and Gender in Research

Laboratory animals	The full list of species used in our study is available in the Supplementary Information. Animals were all lab housed, and ultimately trace their origin back to wild caught in the Lake within a minimal number of generations.
Wild animals	N/A
Reporting on sex	Results are reported on the whole, and sex information is available in the supplementary data.
Field-collected samples	N/A
Ethics oversight	All experiments were performed under holding permit nrs. 1010H and 1035H, and experimental permit nrs. 2356 and 3102 issued by the cantonal veterinary office Basel.

Note that full information on the approval of the study protocol must also be provided in the manuscript.

Plants

Seed stocks	N/A
Novel plant genotypes	N/A
Authentication	N/A
